# Leopard syndrome: the potential cardiac defect underlying skin phenotypes

**DOI:** 10.1186/s41065-021-00199-5

**Published:** 2021-09-06

**Authors:** Xiaojie Yue, Xiong Zhao, Yefeng Dai, Lan Yu.

**Affiliations:** 1 Department of Burn and Plastic Surgery, The Children’s Hospital, Zhejiang University School of Medicine, National Clinical Research Center for Child Health, No. 3333, Binsheng Road, Binjiang District, Hangzhou, Zhejiang China; 2 The Children’s Hospital, Zhejiang University School of Medicine, National Clinical Research Center for Child Health, No. 3333, Binsheng Road, Binjiang District, Hangzhou, Zhejiang China

**Keywords:** LEOPARD syndrome, PTPN11 mutation, Multiple lentigines, Acquired melanocytic nevi

## Abstract

LEOPARD syndrome (OMIM #151,100) caused by a germline PTPN11 mutation are characterized as multisystemic anomalies and variable marked phenotypes such as multiple lentigines and cafe´-au-lait spots, electrocardiographic conduction abnormalities, ocular hypertelorism/obstructive cardiomyopathy, pulmonary stenosis, abnormal genitalia, retardation of growth, and deafness. Phenotype overlap complicates clinical discrimination within RASopathies, making the diagnosis of LEOPARD more confusing and challenging. Besides, LEOPARD patients do not usually present with all these typical clinical features, increasing the possibility of underdiagnosis or misdiagnosis.

Herein, we report a case of LEOPARD syndrome in a patient who only presented with pigmented skin spots and was initially diagnosed with multiple acquired melanocytic nevi. Subsequent pathological examination confirmed the diagnosis of multiple lentigines rather than melanocytic nevi. A genetic study showed a germline PTPN11 (Tyr279Cys) mutation and raised the suspicion of LEOPARD syndrome. A subsequent ECG examination detected potential cardiac defects and confirmed the diagnosis of LEOPARD. We considered that the potential damage of other systems underlying the skin multiple lentigines should not be ignored. The diagnosis of LEOPARD syndrome in an early stage before cardiac damage has reached a serious and irreversible stage can be meaningful for patients to fully understand the potential risks, complications and prognosis of the disease and to take appropriate precautions to prevent the potential risk of cardiac damage.

## Background

LEOPARD syndrome (OMIM #151,100), as an extremely rare inherited autosomal dominant disease, is characterized by multisystemic anomalies and variable marked phenotypes and was first reported by Gorlin et al. in 1969 [1]. The acronym of the term LEOPARD stands for the following cardinal manifestations: multiple lentigines and cafe´-au-lait spots, electrocardiographic conduction abnormalities, ocular hypertelorism/obstructive cardiomyopathy, pulmonary stenosis, abnormal genitalia, retardation of growth, and deafness [1–4]. However, LEOPARD patients do not usually present with all these typical clinical features. Clinical phenotypes are diverse, ranging from only atypical lentigines [5] to severe cardiac defects and life-threatening hypertrophic cardiomyopathy, as well as tumors such as malignant melanoma,[6, 7] leading to difficulties in diagnosis [8, 9]. Although considered to represent lentigines, the darkly pigmented patches frequently observed in LEOPARD patients can also be melanocytic nevi based on histological characteristics [10]. In addition, LEOPARD syndrome phenotypic features strongly overlap with those of Noonan syndrome (OMIM #163,950) and neurofibromatosis type 1 (OMIM #162,200), which are syndromes of a group of disorders associated with mutations in the RAS-MAPK pathway, known as RASopathies [11, 12]. Therefore, phenotype overlap complicates clinical discrimination within RASopathies, making the diagnosis of LEOPARD more confusing and challenging. Molecular diagnosis can provide more information and is essential for differential diagnosis. Over 65% of LEOPARD syndrome patients harbor missense mutations in PTPN11 Tyr279Cys and Thr468 Met mutations, while PTPN mutations account for approximately 50% of Noonan syndrome cases [13, 14].

In this study, we reported a case of LEOPARD syndrome in a patient who only presented with pigmented skin spots and was initially diagnosed with multiple acquired melanocytic nevi. Subsequent pathological examination confirmed the diagnosis of multiple lentigines rather than melanocytic nevi. A genetic study showed a germline PTPN (Tyr279Cys) mutation and raised the suspicion of LEOPARD syndrome. A subsequent ECG examination detected potential cardiac defects and confirmed the diagnosis of LEOPARD.

Multisystemic damage hidden by underlying skin lesions should not be ignored and misdiagnosed. As a dermatologist, early recognition and diagnosis of LEOPARD syndrome is essential to prevent, detect, and treat potential multisystemic anomalies, as well as ensuring an improve quality of life. We recommended molecular analysis to facilitate LEOPARD syndrome diagnosis, regular clinical follow-ups and precautions to prevent the potential risk of cardiac damage, and appropriate initial treatment. Interdisciplinary discussion and a multidisciplinary, multimodal approaches are required to achieve complete evaluation. We emphasize the importance of differentiating skin involvement in LEOPARD syndrome and the subsequent monitoring and detection of malignant transformation [6, 7]. Targeted therapy with rapamycin may be beneficial for LEOPARD patients with cardiac involvement [15].

## Case presentation

**Fig. 1:**
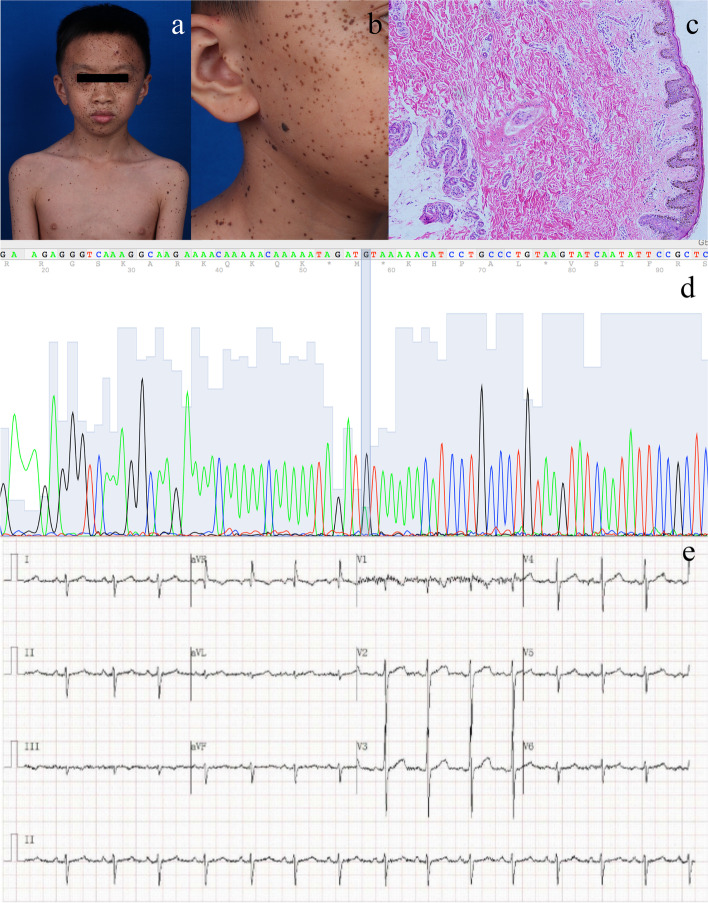
**a** Clinical manifestation of the patient. Multiple pigmented spots were unevenly distributed in different areas of his body, predominantly concentrated on the face, and involved the palpebral conjunctiva. **b** The spots were scattered with various light-to-dark brown colors and heterogeneous sizes less than 3 mm. **c** Histological analysis confirmed the diagnosis of multiple lentigines (hematoxylin–eosin, original magnification × 100). **d** Sanger sequence of germline mutation of PTPN11 (Tyr279Cys, c.836A > G). **e** ECG examination showed extreme right axis deviation (QRS axis: + 232°; Paper speed: 25mm/s; Sensitivity: 10mm/mV)

An eleven-year-old boy came to our dermatology clinic with multiple darkly pigmented spots covering his whole body. The pigmented spots first presented at the age of 5 and gradually increased in number with age. Physical examination revealed that the spots were unevenly distributed in different areas of his body, predominantly concentrated on the face, and involved the palpebral conjunctiva (Fig. 1a). The spots were scattered with various light-to-dark brown colors and heterogeneous sizes less than 3 mm (Fig. 1b). No obvious skeletal abnormalities or dysmorphic features were found during physical examination. According to the patient's parents, family history was unremarkable, and no other systemic diseases were disclosed. The patient was initially diagnosed with multiple acquired melanocytic nevi based on clinical manifestations. Considering that acquired melanocytic nevi are histologically known to have a potential risk of malignancy, we performed a minimally invasive biopsy on one spot (D = 2 mm, all layer biopsy of the skin) under local anesthesia to determine the histological changes and promote further diagnosis after obtaining verbal and written informed consent from the patient’s parents.

The histology results demonstrated features compatible with lentigo simplex: increased number of basal melanocytes and elongation of crests. Histological analysis confirmed the diagnosis of multiple lentigines rather than melanocytic nevi (Fig. 1c).

Since multiple lentigines are usually associated with hereditary syndromes involving multiple organ systems, we proceeded to conduct whole exome sequencing (WES) with the remaining skin tissue after histological analysis.

The molecular study identified a germline mutation in the PTPN11 mutation (Tyr279Cys, c.836A > G) in the tissue sample with a mutation frequency of 44.32% (Fig. 1d). The genetic result raised the suspicion of LEOPARD syndrome. We then performed ECG examination to detect potential cardiac defects and to confirm the diagnosis of LEOPARD syndrome. ECG examination showed extreme right axis deviation (QRS axis: + 232°), suggesting right ventricular hypertrophy (Fig. 1e).

In contrast to the previous diagnosis, based on the multiple lentigines confirmed by histological analysis, a genetic study revealing a germline PTPN11 mutation, and ECG analysis suggesting potential cardiac defects, we diagnosed the child with LEOPARD syndrome.

## Discussion

LEOPARD (OMIM #151,100), also known as neuro-cardio-cutaneous (NCFC) syndrome, is a rare autosomal dominant disease that is characterized by multisystemic disorders and typical germline PTPN11 mutations [2–4, 12, 16]. Approximately 85% of patients with LEOPARD syndrome harbor a missense mutation in the PTPN11 gene, two of which Tyr279Cys and Thr468 Met account for 65% of cases [12]. Germline PTPN11 mutations are responsible for approximately 40–50% of Noonan syndrome cases, leading to a unique phenotypic association between the two syndromes. However, the functional effects of PTPN mutations in these two diseases are completely opposite. In Noonan syndrome, it is a gain-of function mutation, while in LEOPARD, the mutation reduces protein tyrosine phosphatase activity [17, 18]. The symptoms and prognosis of LEOPARD patients are varied and involve a wide spectrum of clinical features with marked variations in expression, including multiple lentigines, cardiac defects, growth retardation effects, facial dysmorphisms, genitalia abnormalities, and deafness [8, 9, 12].

Multiple lentigines are the most distinctive feature that are usually absent in young patients with LEOPARD syndrome. The diagnosis of lentigines was based on personal observation of flat, light-to-dark brown spots by dermatologists. However, skin lesions manifest not only as lentigines but also as melanocytic nevi according to pathological analysis [10, 19]. In addition, malignant melanoma has been reported to occur in in two cases of LEOPARD patients [6, 7]. The activation of the RAS ERK signaling pathway has been implicated in various types of cancer including melanoma, and the suppression of the protein-tyrosine phosphatase SHP-2 encoded by PTPN11 gene favors tumorigenesis due to an alteration of the STAT3 pathway, a pathway involved in the genesis of melanoma. It is not enough to diagnose skin lesions as lentigines only by physical examination since we can easily overlook or neglect melanocytic nevi and the risk of melanoma. Regular dermoscopic examination can yield meaningful information about potential melanoma in these patients. In this study, we performed pathological examination to confirm the diagnosis of multiple lentigines in this patient. We recommend careful physical examination, dermoscopic examination, together with pathological examination, if possible, to determine the nature of the skin lesions and regular follow-up to monitor and detect the potential malignant transformation of melanocytic nevi lesions.

The diagnostic criteria of LEOPARD syndrome include the presence of multiple lentigines and two cardiac features [3]. The absence or mildness of some features and phenotypic overlap with Noonan syndrome and neurofibromatosis type 1 increase the possibility of underdiagnosis or misdiagnosis in some cases with only partial phenotypes. Genetic analysis adds more information for diagnosis and can be beneficial in this difficult circumstance. In this study, the patient presented at our clinic only with multiple lentigines and did not mention any cardiac or hearing impairment. We performed whole exome sequencing (WES) for molecular analysis and electrocardiogram (ECG) to detect possible cardiac defects.

We considered that the potential damage of other systems underlying the skin multiple lentigines should not be ignored. The diagnosis of LEOPARD syndrome in an early stage before cardiac damage has reached a serious and irreversible stage can be meaningful for patients to fully understand the potential risks, complications and prognosis of the disease and to take appropriate precautions to prevent the potential risk of cardiac damage. Laser therapy has been reported to successfully treat lentigines in LEOPARD patients [20]. Moreover, Marin. T. M. et al. reported that rapamycin can reverse hypertrophic cardiomyopathy of PTPN11 mutation-associated LEOPARD syndrome in a mouse model [15]. Molecular analysis and subsequent targeted drugs provide a new approach for treatment. As dermatologists, we recommend interdisciplinary discussion and a multidisciplinary, multimodal approach to achieve complete dermatological, cardiological, genitourinary and neurological examination and evaluation as well as treatment.

## Conclusion

Herein, we report one male patient with multiple pigmented skin spots who was initially diagnosed with multiple acquired melanocytic nevi but was later diagnosed with LEOPARD syndrome (OMIM #151,100) based on a germline PTPN11 mutation. A subsequent ECG examination detected potential cardiac defects. The diagnosis of LEOPARD syndrome in an early stage before cardiac damage has reached a serious and irreversible stage can be meaningful for patients to fully understand the potential risks, complications and prognosis of the disease and to take appropriate precautions to prevent the potential risk of cardiac damage.

## Data Availability

All data used during the study appear in the submitted article.

## Abbreviations

WES: Whole exome sequencing
ECG: Electrocardiogram
